# Supplementary material for: From sequence to enzyme mechanism using multi-label machine learning
Source: BMC Bioinformatics. 2014 May 19;15:150. doi: 10.1186/1471-2105-15-150 (PMC4229970; doi:10.1186/1471-2105-15-150)
Supplement: Additional file 2 — Java code of ml2db. Additional file ml2db_code.tar.gz contains the Java source code to run the multi-label machine learning experiments and save the results to database. The code’s Javadoc is included. [file 1471-2105-15-150-S2.zip › additional file 2/ml2db/ml2db/doc/mulan/core/package-summary.html]

mulan.core


JavaScript is disabled on your browser.


- Overview
- Package
- Class
- Use
- Tree
- Deprecated
- Index
- Help

- Prev Package
- Next Package

- Frames
- No Frames

- All Classes

# Package mulan.core

- Class Summary

  | Class | Description |
  | --- | --- |
  |  |  |
  | --- | --- |
  | MulanJavadoc | This class uses weka's Javadoc auto-generation classes to generate Javadoc  comments and replaces the content between certain comment tags. |
  | Util | Class which provides various utility methods. |
- Exception Summary

  | Exception | Description |
  | --- | --- |
  |  |  |
  | --- | --- |
  | ArgumentNullException | This exception is raised when a null reference is passed to a method that does not accept a null reference for an argument. |
  | MulanException | Represents a root base class for checked exceptions thrown within Mulan library. |
  | MulanRuntimeException | Represents a root base class for unchecked exceptions thrown within Mulan library. |
  | WekaException | The convenience exception, which can be used to wrap up checked general `Exception` commonly thrown by underlying Weka library into anonymous runtime exception. |

- Overview
- Package
- Class
- Use
- Tree
- Deprecated
- Index
- Help

- Prev Package
- Next Package

- Frames
- No Frames

- All Classes
